# Modification of Purine and Pyrimidine Nucleosides by Direct C-H Bond Activation

**DOI:** 10.3390/molecules20034874

**Published:** 2015-03-17

**Authors:** Yong Liang, Stanislaw F. Wnuk

**Affiliations:** Department of Chemistry and Biochemistry, Florida International University, Miami, FL 33199, USA; E-Mail: ylian004@fiu.edu

**Keywords:** C-H activation, cross-coupling, direct arylation, nucleosides, purines, pyrimidines

## Abstract

Transition metal-catalyzed modifications of the activated heterocyclic bases of nucleosides as well as DNA or RNA fragments employing traditional cross-coupling methods have been well-established in nucleic acid chemistry. This review covers advances in the area of cross-coupling reactions in which nucleosides are functionalized via direct activation of the C8-H bond in purine and the C5-H or C6-H bond in uracil bases. The review focuses on Pd/Cu-catalyzed couplings between unactivated nucleoside bases with aryl halides. It also discusses cross-dehydrogenative arylations and alkenylations as well as other reactions used for modification of nucleoside bases that avoid the use of organometallic precursors and involve direct C-H bond activation in at least one substrate. The scope and efficiency of these coupling reactions along with some mechanistic considerations are discussed.

## 1. Introduction

Transition metal catalyzed traditional cross-coupling reactions have contributed significantly to the formation of new carbon-carbon bonds and to the synthesis of biaryl compounds. With few exceptions, the traditional Pd-catalyzed coupling reactions require two activated substrates, one is the organometallic, alkene (Heck reaction), or terminal alkyne (Sonogashira reaction) and the other is the halide or triflate [1,2]. The most often used Stille, Suzuki, Negishi, Kumada and Hiyama reactions need an organometallic (Sn, B, Zn, Mg, and Si) component and a halide or pseudohalide. Owing to the high impact of these reactions in organic synthesis, natural product synthesis and pharmaceutical applications, the 2010 Nobel Prize in Chemistry was awarded jointly to Richard F. Heck, Ei-ichi Negishi and Akira Suzuki [3]. Pd-catalyzed cross-coupling reactions are carried out under mild conditions and can be performed in the presence of most functional groups. The mechanisms in most cases follow three major steps of: (*i*) oxidative addition, (*ii*) transmetallation, and (*iii*) and reductive elimination [1,2].

Transition metal-catalyzed cross-coupling reactions which are based on direct C-H functionalization have been recently developed [4,5,6,7,8,9]. These methodologies, which eliminate the use of organometallic substrates, compete with traditional Pd-catalyzed cross-couplings in the development of new strategies for the formation of carbon-carbon bonds. These reactions require only one activated substrate (C-H activation) and sometimes even no activation is required for either substrate (double C-H activation). They are atom efficient and avoid the synthesis of often unstable activated substrates. Major challenges associated with C-H functionalization reactions include: (*i*) the need for developing regioselective activation of specific C-H bonds in the presence of other C-H bonds; (*ii*) low chemoselectivity which means it is necessary to protect sensitive functional groups before performing the coupling; and (*iii*) the necessity to work at high temperature needed to activate C-H bonds with intrinsic low activity, which often causes decomposition of the substrates. Pd and Cu are two of the most common transition metal catalyst used for the C-H functionalization.

**Figure 1:**
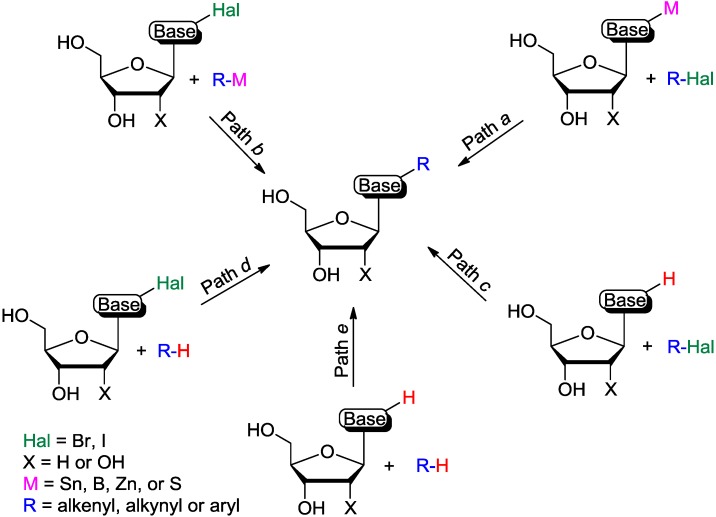
Transition metal catalyzed cross-coupling approaches towards the synthesis of base-modified nucleosides.

Transition metal-catalyzed approaches towards the synthesis of base-modified nucleosides can be divided into five major categories as depicted in Figure 1. The first two approaches are based on cross-couplings between two activated components. One involves reactions between metal-activated nucleoside bases and halides (Figure 1, Path *a*) while the second employs couplings between halo (or triflate) modified nucleoside bases and organometallics (Path *b*). These approaches were extensively reviewed [10,11,12] and are not discussed in this account. The next two approaches are based on cross-couplings between only one activated component and require C-H activation at the second substrate. One involves reactions between C-H activated bond in nucleoside bases and halides (Path c), while the second employs couplings between halo-modified nucleoside bases and arenes, which, in turn, require selective C-H activation (Path *d*). The last approach involves cross-couplings between two inactivated substrates [cross-dehydrogenative coupling (CDC) reactions; Path *e*]. Direct C-H functionalization approaches (Paths *c-e*) alleviate some drawbacks associated with the synthesis of modified nucleosides employed in traditional Pd-catalyzed cross-coupling reactions (Paths *a-b*). They also avoid usage of the toxic organotin components, which are problematic during biological studies, or the sometimes unstable organoboronic substrates.

**Figure 2:**
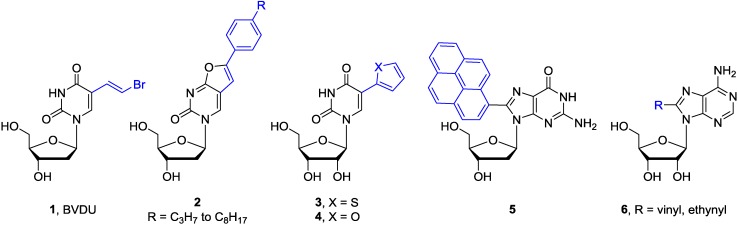
Selected base-modified pyrimidine and purine nucleosides.

Numerous C5 or C6 modified pyrimidine nucleosides and C2 or C8 modified purine nucleosides have been synthesized in last 40 years employing the transition-metal assisted cross-coupling reactions [10]. Some of them show potent biological activity and/or are utilized as mechanistic or labelling probes (Figure 2). For example, the (*E*)-5-(2-bromovinyl)-2′-deoxyuridine (**1**, BVDU) has been found to be a highly potent and selective anti-herpes agent [13]. The bicyclic furanopyrimidine-2-one nucleoside analogues bearing an aryl side chain **2** display remarkable antiviral potency against the Varicella-Zoster virus [14]. The 5-thienyl- **3** or 5-furyluridine **4** were used as molecular beacons for oligonucleotide labeling [15,16,17,18]. The 8-pyrenyl-2'-deoxyguanosine **5** serves as a probe for the spectroscopic study of the reductive electron transfer through DNA [19,20]. Furthermore, the 8-vinyl and 8-ethynyladenosines **6** show cytotoxic activity against tumor cell lines [21], while oligodeoxynucleotides modified with the 8-alkynyl-dG possess thrombin inhibitory activity [22].

## 2. Direct Activation of C8-H Bond in Purine and Purine Nucleosides

### 2.1. Cross-Coupling of Adenine Nucleosides with Aryl Halides

**Scheme 1:**
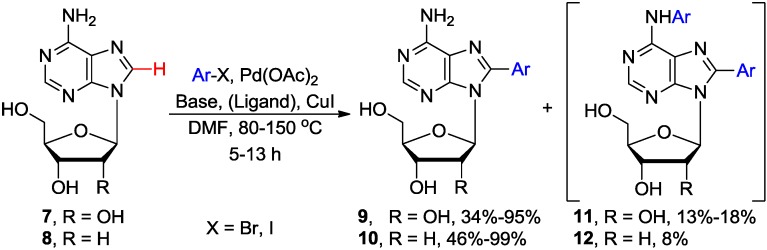
Pd-catalyzed direct C8-H arylation of adenosine **7** and 2'-deoxyadenosine **8** with aryl halides.

Hocek and coworkers reported the first example of direct arylation of adenosine **7** with aryl halides by selective activation of the C8-H bond which gave access to 8-arylated adenosine analogues **9**. The cross-coupling occurred in the presence of a stoichiometric amount of CuI (3 equiv.) and a catalytic load of Pd(OAc)_2_ (5 mol %) in DMF at elevated temperature (100 °C/22 h or 150 °C/5 h) to produce **9** in 50%–68% yields (Scheme 1, Table 1 entry 1) [23]. The authors were able to improve the coupling conditions (e.g., shortening reaction time and lowering the reaction temperature), as compared to their earlier work on C8-H arylation of purines and adenines [24,25] (*vide infra*), by addition of piperidine to the reaction mixture. They assumed [23] that formation of dimethylamine, as a side product of the prolonged heating of the DMF solvent during the C8-H arylation of purines, favorable influenced the rate of the arylation reaction, which is consistent with Fairlamb’s findings [26,27]. Consequently, they found that the addition of higher boiling secondary amine such as piperidine (4 equiv.) was beneficial to the coupling reactions. Couplings of **7** with aryl iodines also produced N6,8-diarylated byproducts **11** in 12%–18% yield, whereas only 8-arylated products **9** were isolated when less reactive aryl bromine were employed.

**Table 1:** Effect of different bases on Pd-catalyzed direct C8-H arylation of adenosine **7** and 2'-deoxyadenosine **8** with aryl halides.

Entry	Base/Ligand	Substrates	Ar	Temp. (°C)	Time (h)	Products	Yield (%)	Reference
1	Piperidine	**7**	4-Tol-I	150	5	**9**	68	[23]
2	Piperidine	**8**	4-Tol-I	125	5	**10**	31	[23]
3	Cs_2_CO_3_	**8**	Ph-I	80	13	**10**	84	[27]
4	Cs_2_CO_3_/Pyridine	**7**	Ph-I	120	13	**9**	30–95 *^a^*	[27]
5	Cs_2_CO_3_/Piperidine	**8**	4-Tol-I	80	15	**10**	85	[26]

*^a^* The yield depends on the substitution at the pyridine ring.

When 2'-deoxyadenosine **8** was subjected to this direct arylation protocol desired 8-arylated products **10** were produced only after the temperature was lowered to 125 °C (31% after 5 h; entry 2). It is worth noting that this protocol was applicable to unprotected nucleosides and allowed for the first time the single-step introduction of the aryl group at the C8 position without the need to (*i*) halogenate nucleoside substrates, or (*ii*) use expensive arylboronic acids or toxic arylstannanes [10].

Fairlamb and coworkers independently developed a Pd-catalyzed direct C8-arylation of adenosine **7** with aryl iodine in the presence of Cs_2_CO_3_ as a base (instead of piperidine), Pd(OAc)_2_ and 3 equiv. of CuI (DMF/120 °C/13 h) to give **9** in good to high yields (Table 1, entry 3) [27]. In addition to **9**, small quantities (~3%) of the N6-arylated byproducts (e.g., **11**) were also produced. Coupling of **7** with 0.5 equiv of 1,4-diiodobenzene yielded 1,4-di-(8-adenosinyl)benzene, albeit in low yield. The less stable 2'-deoxyadenosine **8** could also be arylated under these conditions but the synthesis required lower temperature (80 °C/13 h; 84%) to avoid substantial deglycosylation, which was observed at 120 °C. The authors also found that microwave heating was ineffective due to significant decomposition. However addition of the pyridine substituted with an EWG (e.g., 3-nitropyridine) provided 8-arylated product **9** in up to 95% yield (Table 1, entry 4). It was hypothesized that the electron-deficient pyridines can stabilize active Pd(0) species and increase the reactivity (electrophilicity) of the Pd(II) species and that their beneficial effect is substrate dependent [27]. The direct C8-H arylation of 2'-deoxyadenosine **8** with aryl iodides catalyzed by Pd-nanoparticles (60 °C, 15 h) to give access to 8-arylated product **10** (50% yield) have been recently reported [28].

**Scheme 2:**
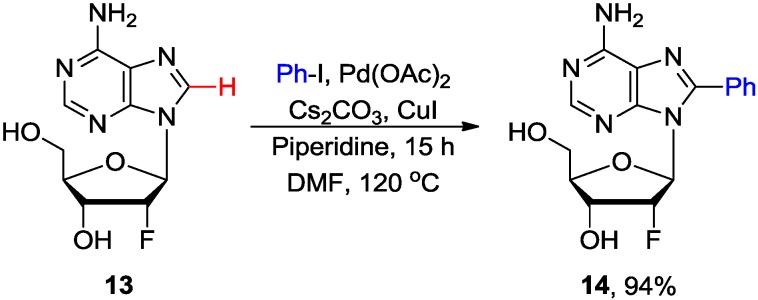
Pd-catalyzed direct C8-H arylation of 2'-deoxy-2'-fluoroadenosine **13** with iodobenzene.

**Scheme 3:**
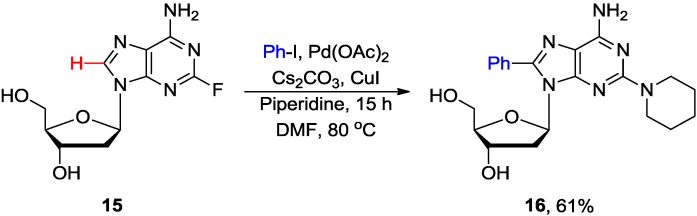
Pd-catalyzed direct arylation of 2-fluoro-2'-deoxyadenosine **15** with iodobenzene.

Fairlamb and coworkers also demonstrated that a combination of a stoichiometric amount of Cs_2_CO_3_ with a substoichiometric amount of piperidine provided the best yield for Pd/Cu-mediated C8 arylation of 2'-deoxyadenosine **8** with various aryl halides (80 °C, 15 h) to give **10** in 32%–95% yields (Table 4 entry 5) [26]. They also noted that sequential direct arylation of **8** with iodo(bromo)benzene followed by Suzuki-Miyaura cross-coupling of the resulting 8-bromophenyl-2'-deoxyadenosine gave convenient access to the new class of rigid organofluorescent nucleosides (RONs) analogues [29]. The arylation conditions were also extended to the adenosine analogues modified at either the ribose or the adenine moieties. Thus, 2'-deoxy-2'-fluoroadenosine **13** gave the 8-arylated product **14** almost quantitatively (94% isolated yield) under similar conditions; probably because the 2'-fluoro substituent is known to increase the stability of the *N-*glycosylic bond and to favor the *syn* conformation (Scheme 2). Coupling of 2-fluoro-2'-deoxyadenosine **15** with iodobenzene also effected 8-arylation concomitant with the displacement of fluorine by piperidine to give the 2,8-disubstituted 2'-deoxyadenosine **16** (Scheme 3) [26]. The chemistry of these couplings has been discussed in recent reviews [11,30].

**Scheme 4:**
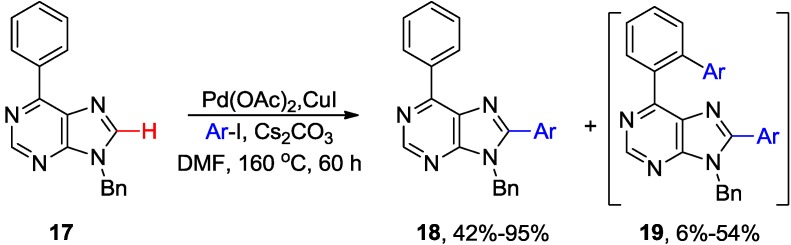
Pd/Cu-mediated direct C8-H arylation of 6-phenylpurines with aryl halides.

These Pd-catalyzed/Cu-mediated methodologies were successfully applied to the synthesis of numerous 8-arylated purines and adenines. In 2006, Hocek and coworkers elaborated the original protocol for the efficient direct C8-H arylation of 6-phenylpurine analogues **17** using aryl iodides in the presence of Cs_2_CO_3_ and CuI to give **18** (Scheme 4) [24]. This route required prolonged heating at high temperature (160 °C/60 h) in DMF. Furthermore, it was essential to perform the coupling with strict exclusion of air to avoid formation of two byproducts, sometimes, in substantial yields (6%–54%). One byproduct was **19**, which was formed by double arylation at C8 position and the *ortho* position of the phenyl ring at C6. The other byproduct was the 8,8'-bispurine dimer. Various 6,8,9-trisubstituted and 2,6,8,9-tetrasubstituted purine analogues were synthesized using this approach in combination with Suzuki cross-coupling reaction and Cu-catalyzed *N*-arylation at 9 position [24,25]. These conditions (Cs_2_CO_3_ or piperidine) were successfully employed for the synthesis of 8,9-disubstituted adenines but 6-*N-*(di)arylated byproducts were observed [24,25]. This protocol was also applied for direct C8-H arylation of adenines anchored to solid phase via 6-*N* amino group (in the presence of piperidine as base) [31].

**Scheme 5:**
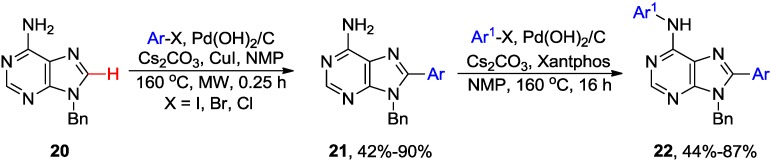
Microwave-assisted direct C8-H arylation of 9-*N*-benzyladenine with aryl halides.

Alami and coworkers developed a microwave-assisted direct C8 arylation of free-(NH_2_) 9-*N-*protected adenine **20** with aryl halides catalyzed by Pd(OH)_2_/C (Pearlman's catalyst) in the presence of CuI (Scheme 5) [32]. Reaction took only 15 min at 160 °C in NMP solvent when Cs_2_CO_3_ was used as base to give **21** in up to 90% yield. The application of Pd(OH)_2_/C catalyst allowed coupling with aryl bromides and even less reactive aryl chlorides [33]. Sequential combination of C8-arylation with ArCl and 6-*N*-arylation with ArBr or ArI (using Xantphos [34] instead of CuI) provides access to disubstituted adenines **22** [33].

**Scheme 6:**
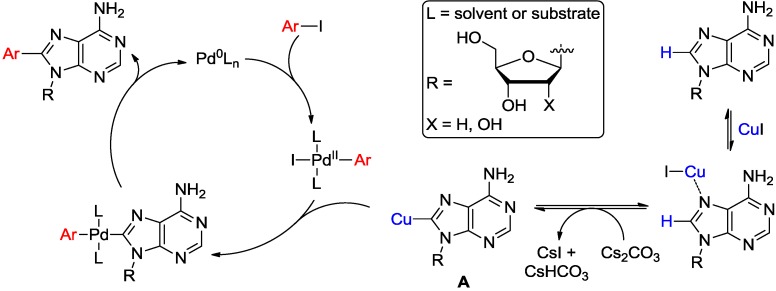
Proposed mechanism for the direct arylation at C8 position of adenosine [26,27].

Fairlamb and coworkers reported a detailed mechanism for the direct C8-arylation of adenine ring with aryl halides mediated by Pd and Cu in the presence of Cs_2_CO_3_ [26,27]. The authors noted that the use of a stoichiometric amount of Cu(I) is key to the direct arylation of the adenine ring and that the process parallels the arylation of imidazole ring at the 2 position [35]. As depicted in Scheme 6, Cu(I) was proposed to assist the C-H functionalization process by an initial coordination to the adenine *N*7 atom. The subsequent base-assisted deprotonation leads to the formation of 8-cuprioadenine intermediate **A** or *N*-heterocyclic carbene like cuprates, which can then undergo a standard Pd(0) catalytic cycle for cross-coupling with aryl halides. This process resembles Sonogashira's reaction between alkynylcuprates and halides [26,27]. The requirement for excess of CuI was attributed to the high binding affinity of Cu(I) for both the substrate and presumably the 8-arylated product(s). The dinucleoside copper(I) complex between 7-N and 6-NH atoms of the adenine have been identified as important intermediate [26].

### 2.2. Cross-Coupling of Inosine and Guanine Nucleosides with Aryl Halides

**Scheme 7:**
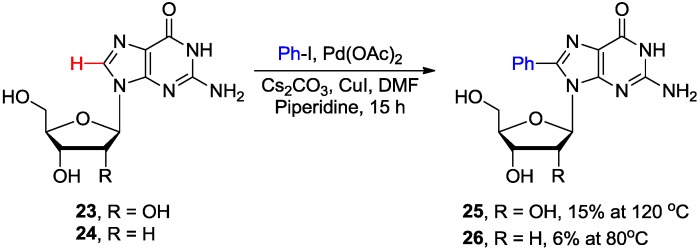
Direct C8-H arylation of guanosine **23** and 2'-deoxyguanosine **24** with iodobenzene.

Guanosine **23** was found to be a poor substrate for the direct C8 arylation as indicated by the low yield (15%) of 8-phenylated product **25** under the conditions (120 °C) which were effective for adenosine analogues (Scheme 7) [26]. Analogous arylation of 2'-deoxyguanosine **24** at lower temperature (80 °C) yielded product **26** but only in 6% yield. The authors hypothesized that in the case of guanine substrates, Cu^I^-coordination most probably occurs at sites distal to C8 hampering efficient arylation. A similar inhibitory effect, associated with the ionizable protons in the guanine moiety was observed during the Suzuki couplings with 8-haloguanine nucleosides [36]. The authors also suggested that guanine-type nucleosides are poor substrates for direct C8-H arylation due to the lack of the "templating" role of exocyclic 6-amino group present in adenine nucleosides.

**Scheme 8:**
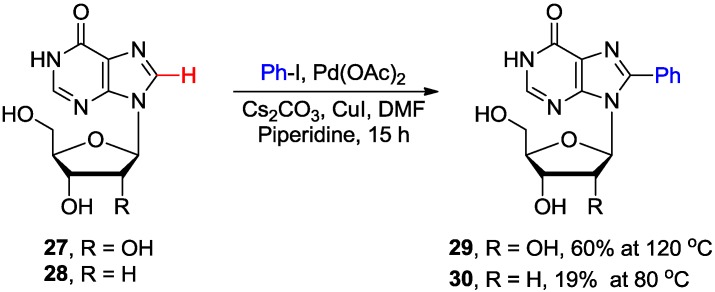
Direct C8-H arylation of inosine **27** and 2'-deoxyinosine **28** with iodobenzene.

The Pd-catalyzed/Cu-mediated direct C8-H arylation of inosine **27** proceeded proficiently to afford 8-phenylated product **29** in good yield (60%) at 120 °C (Scheme 8) [26]. The analogous functionalization of 2'-deoxyinosine **28**, due to the stability of the glycosylic bond, had to be carried out at lower temperature to give product **30** but in only 19% yield.

**Scheme 9:**
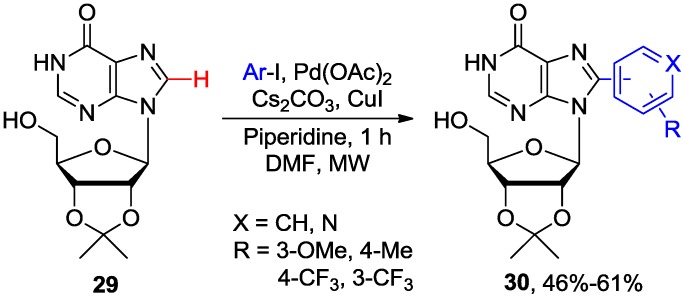
Microwave-assisted direct C8-H arylation of inosine **29** with aryl iodides.

Recently, Pérez and coworkers synthesized the 8-arylated inosine analogues via a microwave-assisted Pd/Cu-catalyzed direct C8-H arylation [37]. In order to increase the solubility of the nucleoside substrate, 2',3'-*O*-isopropylideneinosine **29** was employed to couple with iodopyridines or aryl iodides, by adopting Fairlamb’s protocol [26,29], to produce **30** in only 1 h at 120 °C (Scheme 9).

### 2.3. Synthesis of Fused Purines *via* Inter- or Intramolecular Direct C8-H Arylation

**Scheme 10:**
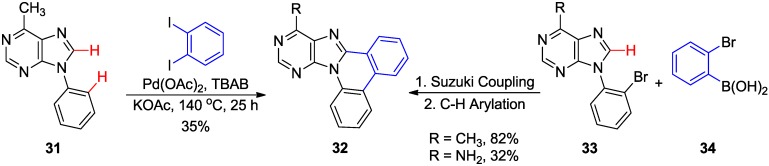
Pd-catalyzed cyclization of 9-*N*-arylpurines via C8-H activation.

Hocek and coworkers developed a direct C8-H arylation of 9-*N*-phenylpurine **31** for the synthesis of fused purine analogues of type **32** with *e*-fusion (position 8 and 9 of purine ring). Thus, Pd-catalyzed intermolecular double direct C-H arylation of 6-methyl-9-*N-*phenylpurine **31** with 1,2-diiodobenzene gave **32** (R = CH_3_) in modest yield (35%). Alternatively, the sequential Suzuki coupling of 9-(2-bromophenyl)adenine **33** (R = NH_2_) with 2-bromophenylboronic acid **34** followed by intramolecular C8-H arylation also gave the desired product **32** (R = NH_2_) in moderate to high yields which preserves the base-pairing and major groove facets of the intact adenine ring (Scheme 10) [38]. However, attempted intramolecular oxidative coupling of 8,9-diphenyladenine failed to give **32**.

**Scheme 11:**
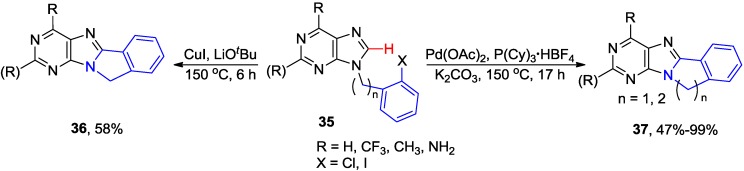
Pd/Cu-catalyzed intramolecular cyclization of 9-*N*-substituted purines via C8-H activation.

The purines **37** with five or six-membered e-fused rings were also synthesized by intramolecular cyclizations of 9-(2-chlorophenylalkyl)purines **35** (n = 1 or 2; X = Cl) employing conditions developed by Fagnou [39] for direct arylation with aryl halides [Pd(OAc)_2_/tricyclohexylphoshine/K_2_CO_3_ in DMF] (Scheme 11) [38,40]. Domínguez and coworks reported the synthesis of five-membered ring analogue **36** by Cu-catalyzed direct C8-H arylation of 9-(2-iodophenylmethyl)purines **35** (n = 1, X = I) in 58% yield [41].

**Scheme 12:**
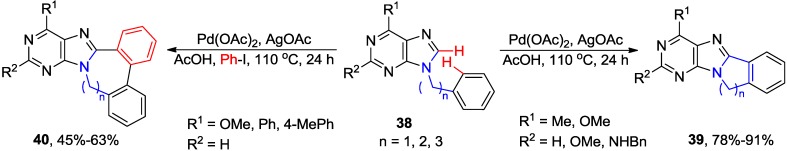
Pd-catalyzed intramolecular cross-dehydrogenative arylation of 9-*N-*substituted purines via C8-H bond activation.

The five-, six- or seven-membered *e-*fused purines **39** have been prepared by the intramolecular double C-H activation (at C8 and *ortho* position of the phenyl ring) of 9-*N-*phenylalkylpurines **38** in the presence of Pd catalyst and silver salt oxidant in high to excellent yields (Scheme 12) [42]. The seven-, eight-, or nine-membered *e-*fused purines of type **40** were prepared by the one-pot coupling of **38** with iodobenzene [42]. The reaction sequence was believed to be initiated by direct intermolecular C8-H arylation of **38** with iodobenzene followed the intramolecular cross-dehydrogenative-arylation between two phenyl rings to give product **40**.

### 2.4. Miscellaneous Direct C8-H Functionalizations

**Scheme 13:**
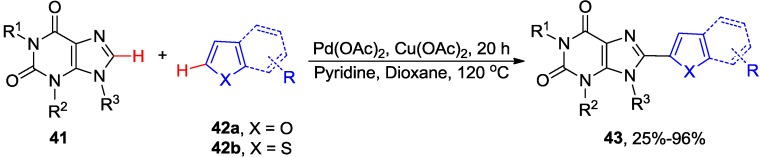
Pd/Cu-catalyzed cross-dehydrogenative arylation of purines with heteroarenes.

You and co-workers reported intermolecular Pd/Cu-catalyzed regioselective C8-H cross-coupling of 1,3-diethyl xanthine **41** (R^1^ = R^2^ = Et, R^3^ = H) with electron-rich furans **42a** and thiophenes **42b** [43]. For example coupling of **41** with 2-methylthiophene in the presence of catalytic amount of the copper salt gave diheteroarene product of type **43** in 96%, indicating the tolerance of the free NH group at the 9 position to the reaction conditions (Scheme 13). The differences in the electron density of two heteroarene components was believed to facilitate the reactivity and selectivity in the two metalation steps [44] of the catalytic cycle of this cross-dehydrogenative arylation reaction.

**Scheme 14:**
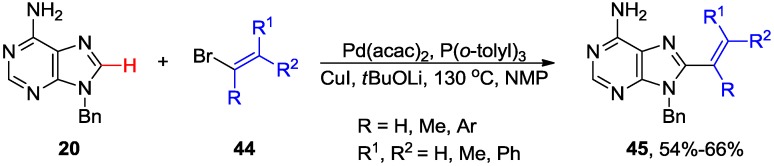
Pd-catalyzed direct C8-H alkenylation of 9-*N-*benzyladenine with alkenyl halides.

The 8-alkenyl adenine analogues **45** have been synthesized via microwave-assisted direct C8-H alkenylation of 9-*N-*benzyladenines **20** with alkenyl bromides **44** (Scheme 14) [32]. Analogous Pd/Cu-mediated C8 alkenylations of 6-(benzylthio)-9-*N*-benzylpurines with styryl bromides provided access to 6,8,9-trisubstituted purines [45]. The optimized conditions (Pd/CuI/*t*BuOLi) were applicable for the selective alkenylation of caffeine, benzimidazole and other aromatic azole heterocycles [45,46]. These are significant developments since it was reported that 8-bromoadenosine was not a good substrate for Mizoroki-Heck reaction [47] making modification at 8 position via direct functionalization of C8-H bond a desirable transformation.

**Scheme 15:**
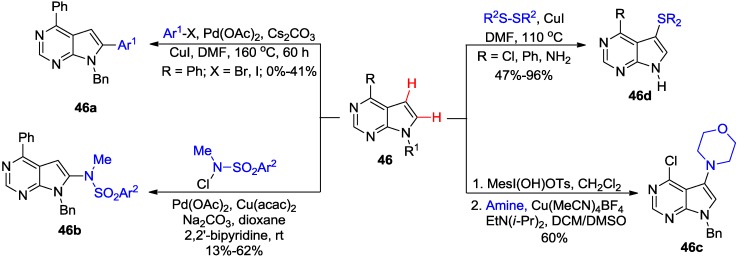
Transition-metal catalyzed direct C-H activation of 7-deazapurines.

Modification of biologically important 7-deazapurines by direct C-H activation have also been explored (Scheme 15). Thus, regioselective Pd-catalyzed direct C8-H arylation of the 6-phenyl-7-deazapurine analogue **46** (R^1^ = Bn) with aryl halides gave corresponding 8-arylated products **46a** albeit in low to moderate yields (0%–41%) [48]. Alternatively, Ir-catalyzed C-H borylation of **46** (R = Ph, R^1^ = Bn) followed by Suzuki coupling with aryl halides afforded **46a** in high yields (79%–95%). Interestingly, Ir-catalyzed C-H borylation was not successful with purines suggesting that the complexation of Ir catalyst to N7 nitrogen might be responsible for the lack of reactivity [48]. The regioselective Pd/Cu-catalyzed direct C8-H amination of the 6-phenyl-7-deazapurine analogue **46** (R^1^ = Bn) with *N*-chloro-*N*-sulfonamides provided the 8-amino-7-deazapurine analogues **46b** [49]. However, subjection of the 6-chloro-7-deazapurine **46** (R^1^ = Bn) to the similar coupling conditions produced a complex mixture. Remarkably, application of conditions, developed by Suna and co-workers for direct C5-H amination of uracils (see Scheme 33), to the same substrate **46** (R = Cl, R^1^ = Bn) provided 7-amino-7-deazapurine analogue **46c** in 60% [50]. Cu-catalyzed direct C-H sulfenylation of 6-substituted-7-deazapurines **46** (R^1^ = H) with aryl or alkyl disulfides provided 7-aryl(or alkyl)sulfanyl products **46d** (47%–96%) in addition to minor quantities of 7,8-bis(sulfanyl) byproducts [51].

## 3. N1-Directed Modifications of C6-Substituted Purine Nucleosides via *ortho* C-H Bond Activation

**Scheme 16:**
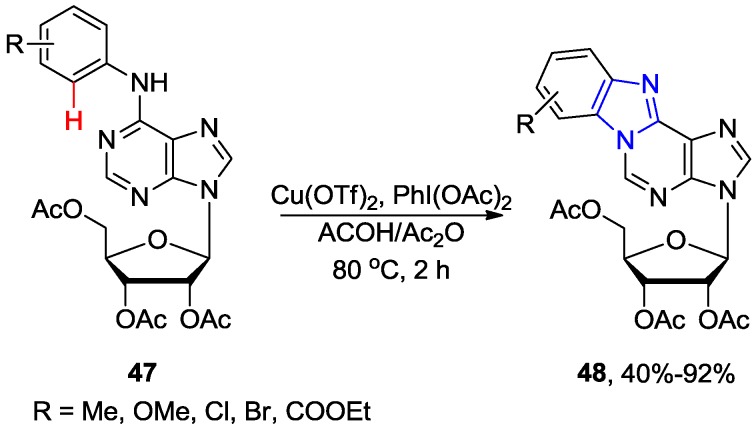
Cu-catalyzed intramolecular direct *ortho* C-H activation/amination of 6-*N*-aryladenosines.

The Cu-catalyzed direct C-H activation/intramolecular amination reaction of the 2',3',5'-tri-*O*-acetyl-6-*N*-aryladenosines **47** were employed for the synthesis of fluorescent polycyclic purine and purine nucleosides of type **48** (Scheme 16) [52]. It was found that addition of Ac_2_O significantly improved the reaction rate (2 h at 80 °C) when Cu(OTf)_2_ (5 mol %) was used as copper source and PhI(OAc)_2_ was used as the oxidant. The 6-*N*-aryladenosines substrates **47** containing electron-withdrawing groups in the benzene ring gave better yields (~85%–92%) than those bearing electron donating groups (~45%–62%). The proposed catalytic cycle involves initial coordination of Cu(OTf)_2_ to the 6-NH····N1 tautomer of substrate **47** at N1 position followed by electrophilic substitution yielding Cu(II) intermediate bridging N1 position of the purine ring and *ortho* position in the aryl ring. Subsequent reductive elimination then provides the fused product **48**.

**Scheme 17:**
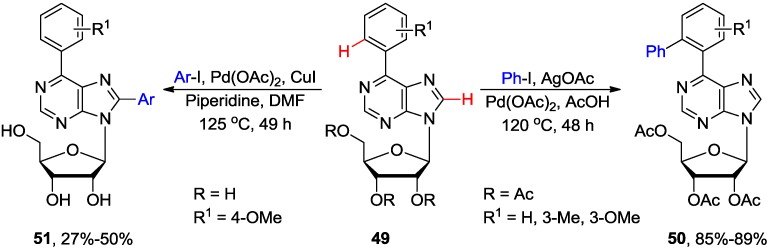
Direct C-H arylation at the *ortho* position in 6-arylpurine nucleosides *vs.* C8-H arylation.

Qu and coworkers developed a Pd-catalyzed strategy for the regioselective *ortho* monophenylation of 6-arylpurine nucleosides (e.g., **49**) via (N1 purine nitrogen atom)-directed C−H activation using a large excess (30 equiv.) of iodobenzene (Scheme 17) [53]. It was essential to perform the reaction under an inert N_2_ atmosphere at 120 °C in the presence of AcOH in order to synthesize **50** in excellent yield (85%). It was believed that AcOH might help (*i*) to overcome the poisoning of the catalyst attributed to the multiple nitrogens present in purine ring, and (*ii*) to facilitate the reductive elimination step. However, the phenylation reaction was not successful when PhI was replaced with PhBr or PhCl.

It is noteworthy that the conditions described by Qu and coworkers (Pd(OAc)_2_/AgOAc/AcOH) are selective for the arylation at the *ortho* site of the C6-phenyl ring, while the CuI-catalyzed coupling of **49** with aryl halides in the presence of Pd(OAc)_2_/piperidine produced exclusively C8-arylated product **51** [23], or a mixture of double arylated products **50**/**51** in addition to 8,8'-dimers when analogous purine substrates **49** and Pd(OAc)_2_/Cs_2_CO_3_ were used [24,25].

**Scheme 18:**
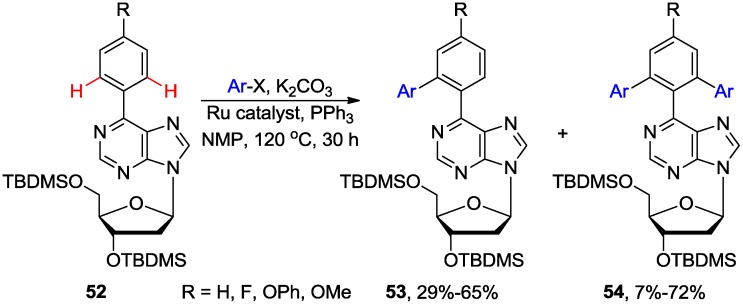
Ru-Catalyzed *N*1-directed *ortho* C-H arylation of 6-phenylpurine nucleosides.

Lakshman and coworkers reported direct arylation of 6-arylpurine nucleosides of type **52** by the Ru-catalyzed C-H bond activation (Scheme 18) [54]. The coupling usually required only 2 equiv. of aryl halides and K_2_CO_3_ or Cs_2_CO_3_ as a base to give mixture of the mono and diarylated *ortho* products **53** and **54** in ratio of approximately of 2–7 to 1 with no 8-arylated byproducts detected. It is noteworthy that these conditions were applicable to acid-sensitive substrates such as 2'-deoxynucleosides as opposed to the previously described Pd-catalyzed arylation promoted by AcOH [53]. Both aryl iodides and bromides gave arylated products in good yield, but aryl iodides proceeded with higher yields and better product ratio. Also, aryl halides bearing EWG gave higher yields compared to the ones bearing EDG.

**Scheme 19:**
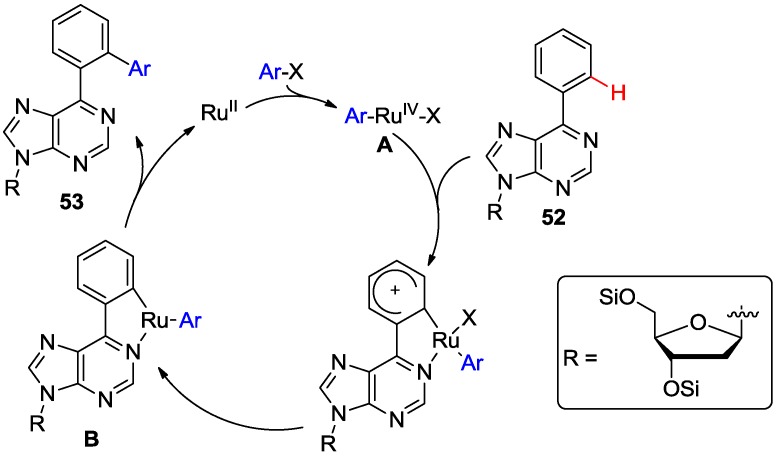
Proposed mechanism for the Ru-catalyzed *N*1-directed C-H bond activation of 6-phenylpurine nucleoside [54].

A possible mechanism for this direct *ortho-*arylation was proposed involving purinyl N1-directed electrophilic attack by the aryl/Ru^IV^ complex **A** on the *ortho* position of the C6-aryl ring atom of the substrate **52** (Scheme 19) [54]. Subsequent reductive-elimination of the five-membered Ru complex **B** gave product **53**. The 2-amino-6-arylpurine nucleosides were unreactive, indicating that the presence of C2-amino group is critical and often inhibits the reactivity of purine nucleoside towards C-H activation reactions.

**Scheme 20:**
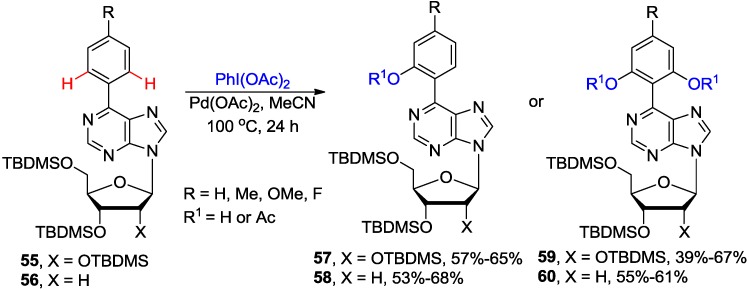
Pd-Catalyzed *N*1-directed *ortho* C-H acetoxylation of 6-arylpurine nucleosides.

The Pd-catalyzed C-H bond activation and oxidation of the silyl protected C6-aryl ribonucleosides of type **55**, as well as the 2'-deoxy counterparts (e.g., **56**), in the presence of PhI(OAc)_2_ as a stoichiometric oxidant in MeCN provided access to monoacetoxylated products **57** or **58** in good yields (Scheme 20) [55]. Increasing the loading of PhI(OAc)_2_ to 3 equiv gave mainly the diacetoxylated products **59** or **60**. The involvement of the N1-purinyl atom in this *N*-directed C-H bond activation was demonstrated by isolation and crystallographic characterization of the dimeric Pd^II^-containing cyclopalladated C6 naphthylpurine derivative. The latter complex, together with PhI(OAc)_2_, was shown to be effective in catalyzing oxidation of substrate **56** to **58**.

**Scheme 21:**
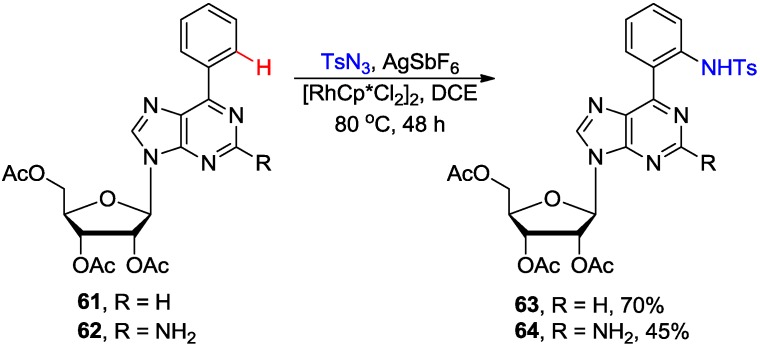
Rh-catalyzed intermolecular direct *ortho* C-H amidation of 6-phenylpurine nucleosides.

Chang and coworkers reported Rh-catalyzed intermolecular amidation of 6-phenylpurine nucleosides with sulfonyl azides via purinyl N1-assisted C-H activation (Scheme 21) [56]. Amidation of 6-arylpurine nucleoside **61** proceeded smoothly and with excellent *ortho-*selectivity to afford product **63** in 70% yield. The presence of a free amino group at the C2 position of purine substrate **62** inhibits once more the coupling efficiency to give amidation product **64** in 45% yield. No glycosylic bond cleavage was noted under the optimized conditions. It is worth noting that the coupling conditions require no additional oxidant and release N_2_ as the only byproduct.

## 4. Direct Activation of C5-H or C6-H Bond in Uracils and Uracil Nucleosides

### 4.1. Cross-Coupling with Aryl Halides

**Scheme 22:**
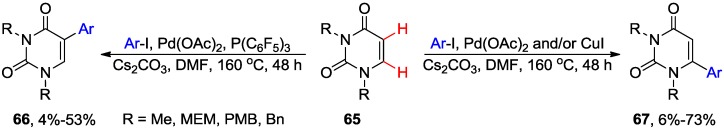
Catalyst-controlled direct C5-H or C6-H arylation of uracil analogues with aryl halides.

Direct C-H arylation of the pyrimidine nucleosides is currently limited to uracil bases. The main challenge which needed to be overcome was a regioselective activation of the C5-H or C6-H bond of the uracil ring. Also lacking are efficient conditions which could be applicable to the natural uridine and 2'-deoxyuridine analogues. Hocek and coworkers reported the regioselective C-H arylations of 1,3-dimethyluracil (**65**, DMU, R = Me) with aryl halides. Thus, arylation in the presence of Pd(OAc)_2_ and Cs_2_CO_3_ mainly formed the 5-arylated uracil analogues **66**, while coupling in the presence of Pd catalyst and CuI (3 equiv.) preferentially formed the 6-arylated derivatives **67** (Scheme 22) [57,58]. Interestingly, Cu-mediated arylation in the absence of Pd catalyst gave exclusively 6-aryluracils **67** albeit in lower yields. These couplings required high temperature (160 °C) and long time (48 h) and were not applicable to the synthesis of unsubstituted uracils. Also the electron-deficient aryl bromides were poor substrates. In order to prepare the unprotected uracil derivatives, these protocols were also applied to *N*-benzyl protected uracil derivatives. Subsequent debenzylation (e.g., **67**, R = Bn) afforded efficiently 5- or 6-aryled uracil derivatives [57,58]. The experimental results indicated that different mechanisms are involved in these diverse arylation reactions [57,58].

**Scheme 23:**
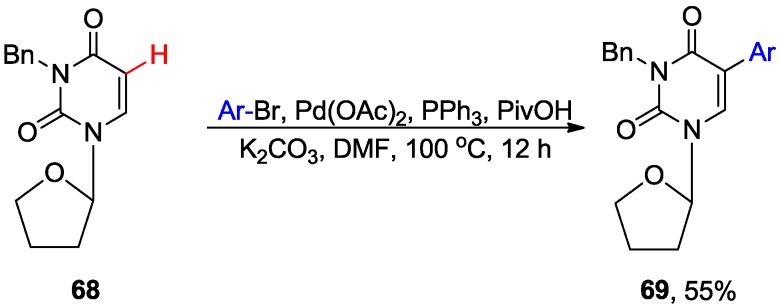
Pd-catalyzed direct C5-H arylation of 1-(tetrahydrofuran-2-yl)-3-benzyluracil.

Kim and coworkers reported the direct arylation of 1,3-dimetyluracil **65** with aryl bromides (including electron-deficient ones) in the presence of Pd(OAc)_2_, K_2_CO_3_ and PivOH (130 °C/12 h/DMF to give predominantly C5 arylated products **66** in up to 79% yield. A small amount of 6-arylated isomers **67** were also observed [59]. However, application of this condition to 1-(tetrahydrofuran-2-yl)-3-benzyluracil **68** (a substrate having a glycosylic bond) resulted in severe decomposition. Nonetheless lowering the temperature to 100 °C (12 h) allowed the isolation of the 5-phenyluracil analogue **69** as the sole product in 55% yield (Scheme 23) [59]. The intramolecular C6-H arylation of the uracil derivatives bearing a Morita-Baylis-Hillman adduct at the N1 position in the presence of TBAB and Pd(OAc)_2_ provided a convenient access to the azepine scaffold [60].

**Scheme 24:**
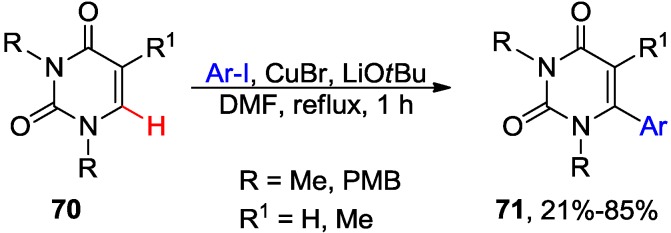
Cu-mediated direct C6-H arylation of 1,3-disubstituteduracil **70** with aryl iodides.

Chien and coworkers further explored the base-dependence of direct activation of uracil C6-H in the presence of Cu catalysts for the synthesis of 6-aryl uracil and 2-aryl-4-pyridone derivatives. The authors found that the CuBr-mediated arylation of DMU **65** (R = H) with aryl iodides (2 equiv.) in the presence of *t*-BuOLi in DMF at reflux gave the C6-arylated derivatives **71** (R^1^ = H) [61]. The 5-substituted DMU analogues **70** (R^1^ = Me) afforded the 5,6-disubstituted derivatives **71** (R^1^ = Me) but in lower yields (Scheme 24). Both N1 and N3 positions in the uracil ring have to be protected for the reactions to proceed.

**Scheme 25:**
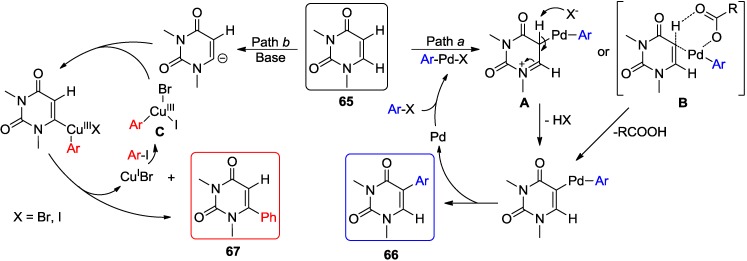
Proposed mechanisms for the regioselective direct arylation of DMU [59,61].

Based on the literature reports, the mechanism for the direct arylation of uracil analogues (e.g., DMU, **65**) with aryl halides at either the C5 or C6 position in the presence of Pd or Cu/(Pd) are summarized in Scheme 25. The coupling with only Pd(OAc)_2_ was proposed to proceed via an electrophilic metalation-deprotonation (EMD) mechanism [5,9] (path *a*, complex **A**), and thus follow the regioselectivity of substitution at the C5 position to give **66** [59]. The formation of the C5 arylated products was also suggested [57] to proceed via the concerted metalation-deprotonation (CMD) mechanism [9,62] (complex **B**). On the other hand, direct C-H arylation in the presence of CuI or in combination with Pd(OAc)_2_ was hypothesized to proceed via an Ullmann-type mechanism [27,63], which involves the formation of a carbanion by the abstraction of the most acidic C6-H [64,65,66] in the uracil ring (e.g., **65**) with base (path *b*). Subsequent cupration, transmetallation and reductive elimination leading to the formation of C6 arylated uracil **67** [57,61]. Alternatively, addition of the copper center of the arylcopper(III) complex **C** to the more nucleophilic C5-position in **65** followed by either base-promoted *anti*-elimination or Heck-type *syn β*-hydrogen elimination has been also proposed for C6-arylation [61]. Moreover, Heck-type carbopalladation of the 5,6-double bond of **65** with ArPdX species has been also considered for the arylation of uracil ring with aryl halides [59].

### 4.2. Cross-Dehydrogenative Coupling with Arenes and Heteroarenes at C5 Position

**Scheme 26:**
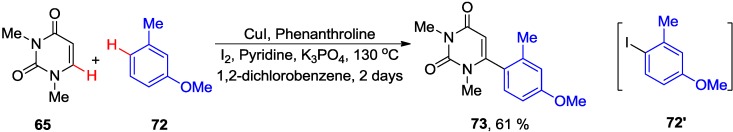
Sequential iodination/direct C6-H arylation of DMU **65** with arenes.

Cross-couplings which required double C-H activation both at uracil and arene substrates have also been developed. Thus, Do and Daugulis reported a highly regioselective CuI/phenantroline-catalyzed oxidative direct arylation of DMU **65** with arenes (e.g., 3-methylanisole **72**) to give C6-arylated uracil **73** as a sole product in 61% yield (130 °C, 48 h) (Scheme 26) [67]. The protocol used iodine as a terminal oxidant and required only a small excess of the arene (1.5–3.0 equiv.). This *overall* cross-dehydrogenative arylation was believed to proceed by *in situ* iodination of one of the coupling components (e.g. 3-methylanisole **72** to give 4-iodo-3-methylanisole **72'**) followed by Cu-catalyzed direct arylation at the most acidic bond in the DMU substrate (C6-H). Recently, an efficient (~70%–80%) and regioselective C-6 arylation of DMU **65** with arylboronic acids in the presence of Pd(OAc)_2_ and ligand (1,10-phenanthroline) at 90 °C for 16 h has been also developed [68]. The coupling failed, however, when unsubstituted uracil or 2',3',5'-tri-*O*-acetyl-3-*N*-methyluridine was used as substrate or when the heteroaromatic boronic acids were employed [68].

**Scheme 27:**
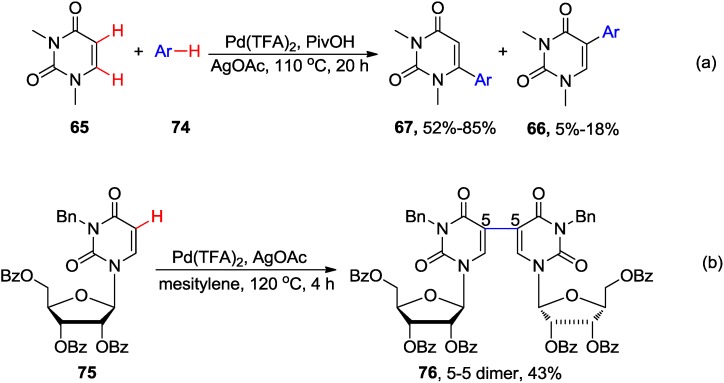
Pd-catalyzed cross-dehydrogenative coupling of uracils and uracil nucleosides.

The Pd-catalyzed cross-dehydrogenative coupling (CDC) of DMU **65** with benzene or xylenes **74** in the presence of PivOH and AgOAc at reflux was found to produce 6-aryluracil analogue **67** as the major product. Minor quantities of 5-arylated counterpart **66** and uracil dimeric byproducts were also formed (Scheme 27a) [59,69]. It is believed that the 6-arylation occurred via CMD process involving Pd^II^(L)(OPiv) species. Deprotonation occurred at the more acid hydrogen at C6 of uracil ring, followed by a second CMD process to give the 6-arylated uracil product **67**. Interestingly, reaction of DMU **65** with mesitylene led only to the formation of 5,5- and 5,6-DMU dimers. Also, application of this protocol to 2',3',5'-tri-*O*-acetyl-3-*N*-benzyluridine **75** gave only the C5-C5 dimer **76** in 43% yield (Scheme 27b) [69].

**Scheme 28:**
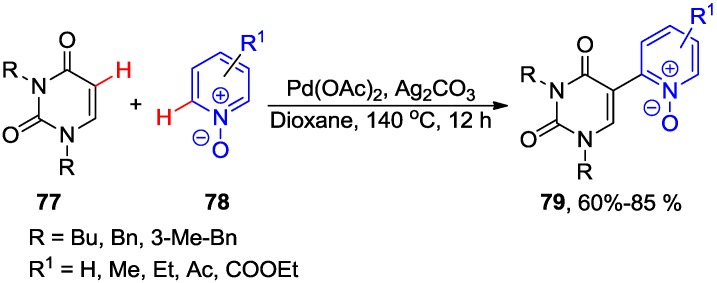
Synthesis of 5-(2-pyridyl-*N*-oxide)uracils **79** via cross-dehydrogenative arylation.

The Pd-catalyzed cross-dehydrogenative heteroarylation between 1,3-dialkyluracils **77** and pyridine-*N*-oxides **78** (3 equiv.) substrates in the presence of Ag_2_CO_3_ at 140 °C for 12 h gave 5-(2-pyridyl-*N*-oxide)uracils **79** in good-to-high yields (Scheme 28) [70]. As expected, the 3-substituted pyridine-*N*-oxides gave products **83** with excellent regioselectivity at the less bulky site. The coupling, however, was not compatible with either 1,3-dibenzoyluracil or unprotected uracil substrates. Reduction of *N-*oxides **79** with PCl_3_ in toluene yielded the corresponding 5-uracil derivatives substituted with a 2-pyridyl ring. The electrophilic palladation at the C5 of uracil and the coordination of the palladium atom to *N*-oxide was believed to control the regioselectivity (at C5 of the uracil ring and C2 of pyridine oxide) of these double C-H activation cross-couplings.

### 4.3. Cross-Dehydrogenative Alkenylation at C5 Position

**Scheme 29:**
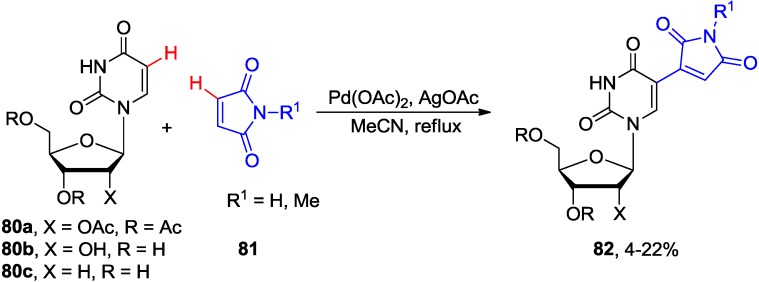
Pd-mediated oxidative coupling of uridine and 2'-deoxyuridine with maleimides.

The discovery of the potent antiviral activity of *E*-5-(2-bromovinyl)-2'-deoxyuridine (**1**, BVDU) led to the exploration of synthetic routes based on the oxidative coupling of uridine nucleosides with alkenes. Such routes avoided the use of mercury that was central to Walker’s synthetic approach involving the condensation of 5-mercurated 2'-deoxyuridine with methyl acrylate and radical decarboxylation-bromination sequence [71]. They also seem advantageous to other coupling protocols which employ coupling between 5-halouracil nucleosides and organometallics or methyl acrylate [10,72]. In 1987, Itahara reported the oxidative coupling of uracil nucleosides **80** with maleimides **81** which gave 5-substituted coupling products of type **82** (Scheme 29) [73]. Using stoichiometric amounts of Pd(OAc)_2_ was however necessary. The yields for uridine and 2'-deoxyuridine substrates were lower (4%–22%) than those of the DMU substrate (~20%–50%). The yields for DMU substrate slightly increased in the presence reoxidants such as AgOAc, Na_2_S_2_O_8_ or Cu(OAc)_2_.

**Scheme 30:**
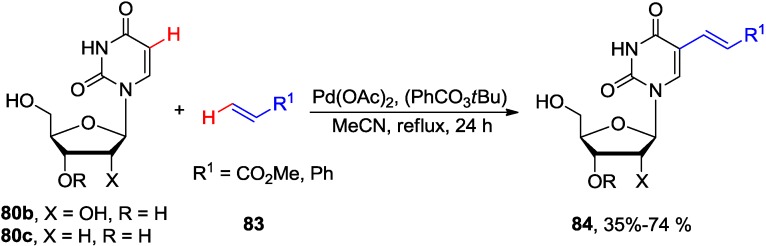
Stoichiometric and catalytic oxidative coupling of uracil nucleosides with methyl acrylate.

Also in 1987, Hirota and coworkers reported the oxidative coupling of uridine **80b** and 2'-deoxyuridine **80c** with methyl acrylate or styrene using either stoichiometric amounts of Pd(OAc)_2_ in MeCN at ambient temperature or catalytic loading of Pd(OAc)_2_ in the presence of *tert-*butyl perbenzoate as the reoxidant to generate 5-vinyl uridine analogues **84** (Scheme 30) [74]. The couplings proceeded stereoselectively to give the *trans* isomers. The reaction conditions were compatible with unprotected and protected uridine substrates, though, coupling of 2',3'-di-*O*-isopropylideneuridine with methyl acrylate produced also the 5',6-cyclouridine byproduct in 23% yield.

**Scheme 31:**
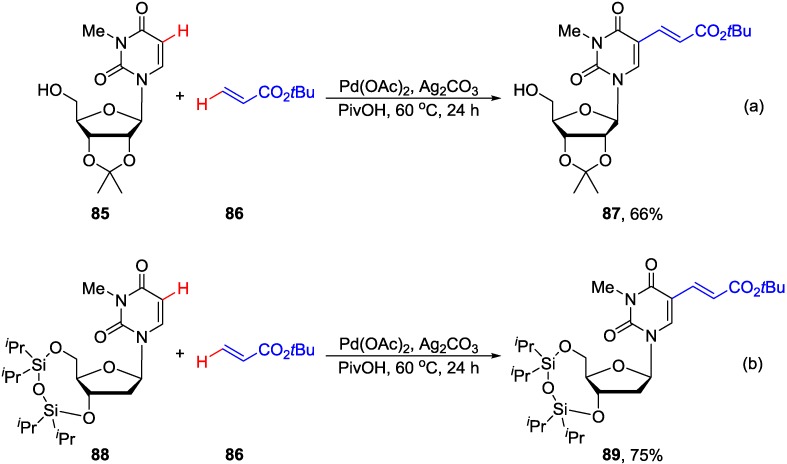
Pd-Catalyzed cross-dehydrogenative alkenylation of uracil nucleosides.

Yun and Georg recently reported Pd-catalyzed cross-dehydrogenative coupling of 1,3-disubstituted uracils as well as protected uridine **85** and 2'-deoxyuridine derivatives **88** with *tert*-butyl acrylate in the presence of AgOAc and PivOH in DMF at 60 °C/24 h to give 5-alkenyl products **87** and **89** in 66% and 75% yield, respectively (Scheme 31) [75]. The coupling occurred with the regio- (C5) and stereoselectivity (*E*-isomer). However, 3-*N*-methyl protection at the uracil ring was necessary. The coupling was postulated to occur via the electrophilic palladation pathway [76] at the C5 position of the uracil ring followed by deprotonation with the pivalate anion to give palladated intermediate. Coordination with the alkenes via transmetallation, and subsequent β-elimination provided 5-alkenyluracil derivatives.

### 4.4. Miscellaneous Direct C-H Functionalizations

**Scheme 32:**
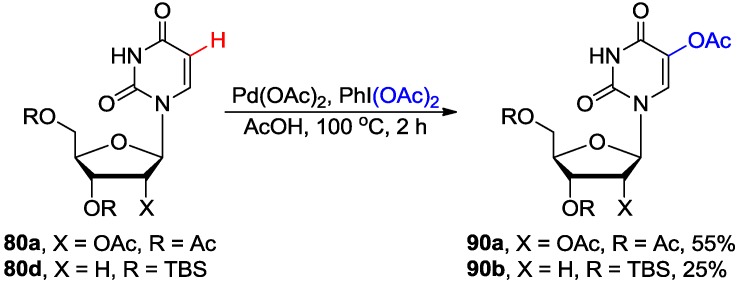
Pd-catalyzed acetoxylation of uracil nucleosides.

Pd-catalyzed direct C-H acetoxylation at the electron-rich C5 position of uracil nucleosides with PhI(OAc)_2_ under reasonably mild conditions (60 °C, 3–5 h) have been also developed (Scheme 32) [77]. The acyl protected uridine **80a** and silyl protected 2'-deoxyuridine **80d** were compatible with these conditions to give the corresponding 5-acetoxy products **90** in 55% and 25% yields, respectively. The reaction was proposed to proceed via oxidative electrophilic palladation at the electron rich C5 position to give the 5-palladauracil intermediate followed by oxidation to give Pd(IV) intermediate, which yielded 5-acetoxyluridine via the reductive elimination of Pd(II).

**Scheme 33:**
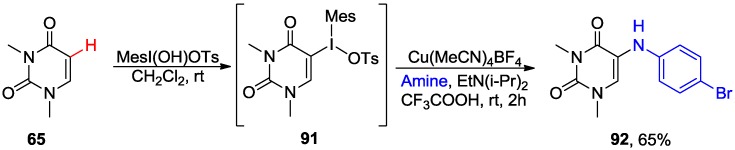
Cu-catalyzed direct C5-H amination of 1,3-dimethyluracil **65**.

The Cu-catalyzed intermolecular C5-H amination of DMU **65** with 4-bromoaniline in the presence of [hydroxy(tosyloxy)iodo]mesitylene has been recently developed (Scheme 33) [50]. The regioselectivity of this C5 amination was proposed to be controlled via the formation of iodonium salt intermediate **91**, which is consistent with C5 electrophilic aromatic substitution that is typical for uracil ring. Subsequent Cu-catalyzed amination gave the 5-amino product **92** in 65% overall yield.

**Scheme 34:**
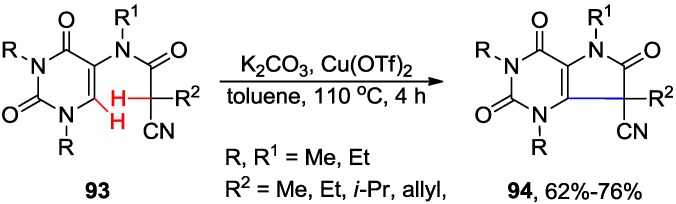
Intramolecular cross-dehydrogenative cyclization at C6 of uracil ring.

Majumdar and coworkers reported the synthesis of pyrrolo[3,2-*d*]pyrimidine derivatives of type **94** by the intramolecular dehydrogenative coupling of the 5-amidouracils **93** via the selective activation of uracil C6-H bond in the presence of Cu(OTf)_2_ (Scheme 34) [78]. This coupling between C_sp2_-H (in the uracil ring) and C_sp3_-H (in the side chain) bonds was not, however, successful when R^2^ = H. The authors suggested that coupling most probably involved single electron transfer (SET) processes and might require a more stable tertiary radical on the side chain to proceed.

**Scheme 35:**
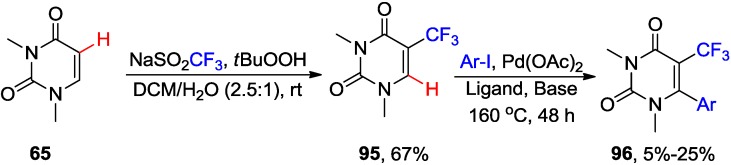
Synthesis of 6-aryl-5-(trifluoromethyl)uracils by direct activation of C5-H and C6-H bonds.

Recently, C5-H trifluoromethylation of DMU **65** by means of electrophilic, nucleophilic or radical “CF_3_” species in an effort to synthesize 6-aryl-5-(trifluoromethyl)uracils (e.g, **96**) by direct activations of both C5-H and C6-H bonds in consecutive manner has been attempted (Scheme 35) [79]. Thus, reactions of **65** with electrophilic (Umemoto's or Togni’s) or nucleophilic (Rupert’s) reagents in combination with Pd or Cu catalyst either failed or led to the formation of 5,5- or 5,6-dimeric products or 5-CF_3_ product in low yields. However, radical trifluoromethylation of **65** with sodium trifluoromethanesulfinate in the presence of *tert*-butyl hydroperoxide provided 1,3-dimethyl-5-(trifluoromethyl)uracil **95** in 67% yield [79]. The subsequent direct arylation at C6-H of **95** with 4-iodotoluene in the presence of Pd(OAc)_2_ and CuI/CsF afforded desired 5,6-disubstituted uracils **96** albeit in low yield (25%); probably because of the electron-withdrawing effect of the CF_3_ group at the 5 position on the pyrimidine ring. This C6-H arylation was not applicable to other aryl halides and often was accompanied by cleavage of the CF_3_ group due to hydrolysis followed by decarboxylation, especially when Cs_2_CO_3_ was used as the base.

## 5. Coupling of 5-Halouracil Nucleosides with Arenes and Heteroarenes

**Scheme 36:**
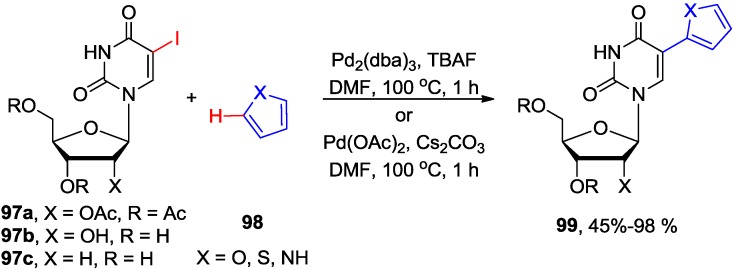
Pd-catalyzed cross-coupling of 5-halouracil nucleosides with arenes and heteroarenes.

The lack of regioselectivity in direct activation of uracil derivatives (C5-H *vs.* C6-H) during cross-couplings with aryl halides, and fact that coupling conditions are usually unsuitable for unprotected uracils and natural nucleosides, were recently overcome by switching the halide substituents from aryl halides to uracil ring and allowing to react of 5-halouridines with arenes instead. Wnuk and coworkers found that the 5-iodouracil nucleosides **97** coupled with simple arenes or heteroaromatics **98** in the presence of Pd_2_(dba)_3_ and TBAF in DMF under milder condition (100 °C/1–2 h) to give the 5-arylated uracil nucleosides **99** in high yields (Scheme 36) [80].

This TBAF-promoted protocol, which proceeded without the necessity of adding ligands and/or additives, worked efficiently with the natural uracils and uracil nucleosides, and was compatible with the stability of the glycosylic bond for 2'-deoxyuridine substrates (e.g., **97c**). The 5-(2-furyl, or 2-thienyl, or 2-pyrrolyl)uridine derivatives **99**, that are important RNA and DNA fluorescent probes [15,16,17,18], were synthesized in up to 98% yields without the necessity of using organometallic substrates. The arylation proceeded also when TBAF was replaced with Cs_2_CO_3_ base with or without the presence of PivOH [80]. The analogous coupling of 5-iodocytidines with arenes failed to afford 5-arylated products.

**Figure 3:**
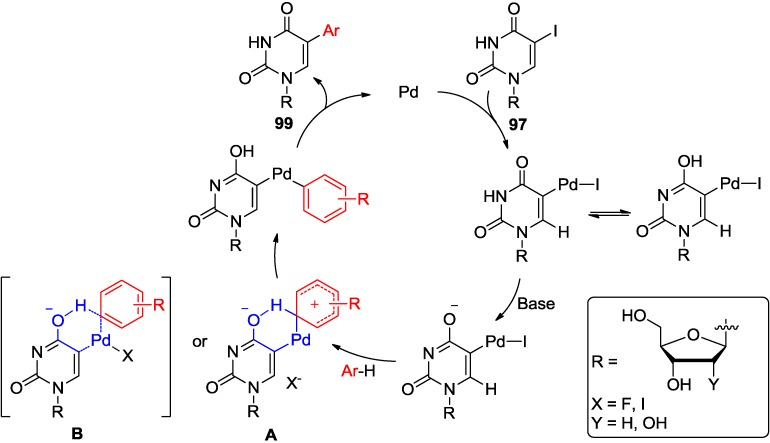
The plausible mechanism for the Pd-catalyzed arylation of 5-iodouridine with arenes.

The fact that 3-*N*-methyl-5-iodouracil substrates, which lack the ability to tautomerize to the enol form, did not undergo these couplings with arenes indicates that the C4-alkoxide (enol form of uracil) may participate in the intramolecular processes of hydrogen abstraction as depicted in Figure 3. The mechanism pathway, after the initial oxidative-addition of palladium to C5-halogen bond, might involve electrophilic aromatic palladation assisted by C4-alkoxide (e.g., **A**) or direct proton abstraction assisted by C4-alkoxide (e.g., **B**) [80]. This would be in agreement with earlier finding [7,81] that direct arylation is facilitated by a Pd-coordinated carboxylate group, which also can assist in intramolecular proton abstraction.

## 6. Conclusions

The use of the Pd(cat.)/Cu(stoich) system in the presence of bases such as Cs_2_CO_3_ and/or piperidine in DMF effects direct regioselective arylation of purine nucleosides at the 8 position. Protected and deprotected adenosine, 2'-deoxyadenosine as well as inosine or guanosine derivatives coupled efficiently under these conditions with aryl halides to give access to 8-arylated products in up to 99% yields. The C8-H functionalization process is proposed to occur via 8-cupriopurine intermediates or *N*-heterocyclic carbene like cuprates, which subsequently can undergo a standard Pd(0)-catalytic cycle for cross-coupling with aryl halides. The cross-dehydrogenative arylation protocols which involve C8-H bond activation have been also developed for the synthesis of purine fused ring systems. Intramolecular N1-purinyl nitrogen directed C-H bond activation was utilized for direct *ortho* modification (arylation, amination or acetoxylation) of the aryl rings at C6 position of purine nucleosides.

In the case of pyrimidine nucleosides, direct arylation protocols have only been developed for uracil bases, which, in turn, usually require protection at the 1-*N* and 3-*N* positions. The biggest challenge how to overcome regioselective C-H activation at the C5 or C6 position of uracil ring have been accomplished by employing different catalysts and ligands. For the cross-coupling with aryl halides, it was found that Pd-based catalytic systems were effective to promote C5 arylation, whereas Pd/Cu or Cu-mediated systems affected C6 arylation with good selectivity. The C5 arylation is proposed to proceed via the electrophilic or concerted metalation-deprotonation mechanisms, while the C6 arylation likely to occur via a cuprate intermediates. Pd-catalyzed cross-dehydrogenative coupling of uracil nucleosides with alkenes or arenes, in the presence of oxidants, were developed to give convenient access to 5-alkenyl/aryl derivatives. The 5-acetoxy and 5-amino derivatives were also synthesized by direct activation of C5-H bond in uracil ring. No examples of couplings involving direct C-H activation of cytosine ring have been reported.

The transition-metal catalyzed syntheses of the modified nucleobases by direct C-H bond activations have been substantially improved in the past decade. However, despite improved coupling efficiency and availability of milder reaction conditions applicable to the less stable deoxynucleosides, application of the direct C-H functionalization approaches to nucleotides and/or short (deoxy)oligonucleotides fragments still will require developing the conditions compatible both with solvent requirements for water-soluble nucleotides and stability of phosphate esters.
